# Identification of a 5-Protein Biomarker Molecular Signature for Predicting Alzheimer's Disease

**DOI:** 10.1371/journal.pone.0003111

**Published:** 2008-09-03

**Authors:** Martín Gómez Ravetti, Pablo Moscato

**Affiliations:** Centre for Bioinformatics, Biomarker Discovery & Information-Based Medicine, The University of Newcastle, Callaghan, Australia; Massachusetts General Hospital and Harvard Medical School, United States of America

## Abstract

**Background:**

Alzheimer's disease (AD) is a progressive brain disease with a huge cost to human lives. The impact of the disease is also a growing concern for the governments of developing countries, in particular due to the increasingly high number of elderly citizens at risk. Alzheimer's is the most common form of dementia, a common term for memory loss and other cognitive impairments. There is no current cure for AD, but there are drug and non-drug based approaches for its treatment. In general the drug-treatments are directed at slowing the progression of symptoms. They have proved to be effective in a large group of patients but success is directly correlated with identifying the disease carriers at its early stages. This justifies the need for timely and accurate forms of diagnosis via molecular means. We report here a 5-protein biomarker molecular signature that achieves, on average, a 96% total accuracy in predicting clinical AD. The signature is composed of the abundances of IL-1α, IL-3, EGF, TNF-α and G-CSF.

**Methodology/Principal Findings:**

Our results are based on a recent molecular dataset that has attracted worldwide attention. Our paper illustrates that improved results can be obtained with the abundance of only five proteins. Our methodology consisted of the application of an integrative data analysis method. This four step process included: a) abundance quantization, b) feature selection, c) literature analysis, d) selection of a classifier algorithm which is independent of the feature selection process. These steps were performed without using any sample of the test datasets. For the first two steps, we used the application of Fayyad and Irani's discretization algorithm for selection and quantization, which in turn creates an instance of the (alpha-beta)-k-Feature Set problem; a numerical solution of this problem led to the selection of only 10 proteins.

**Conclusions/Significance:**

the previous study has provided an extremely useful dataset for the identification of AD biomarkers. However, our subsequent analysis also revealed several important facts worth reporting:

1. A 5-protein signature (which is a subset of the 18-protein signature of Ray *et al.*) has the same overall performance (when using the same classifier).

2. Using more than 20 different classifiers available in the widely-used Weka software package, our 5-protein signature has, on average, a smaller prediction error indicating the independence of the classifier and the robustness of this set of biomarkers (i.e. 96% accuracy when predicting AD against non-demented control).

3. Using very simple classifiers, like Simple Logistic or Logistic Model Trees, we have achieved the following results on 92 samples: 100 percent success to predict Alzheimer's Disease and 92 percent to predict Non Demented Control on the AD dataset.

## Introduction

Recently, Ray *et al.* [1] made a significant contribution to the quest of finding a superior molecular test for an earlier diagnosis of Alzheimer's disease (AD). The method appears to have significantly improved on the state-of-the-art and, as a consequence, their results attracted immediate worldwide attention. Using the abundance of 120 signalling proteins on a training set of 83 archived plasma samples, they produced an 18-protein signature. On two separate test sets of 92 (“AD” Alzheimer's samples against control) and 47 (“MCI” mild cognitive impairment samples) the signature was able to show an overall effectiveness of 81% and 91% for AD predictability.

We started this project by analysing the dataset made available and we are glad to report that we have been able to perfectly reproduce their mathematical methods and results from the available datasets. However, our subsequent analysis also produced several important facts worth reporting: using an integrative bioinformatics approach, we identified a 6-protein signature that halves the number of errors in prediction of the previously proposed signature (on the “AD” dataset.), when using the same classifier (PAM). A 5-protein signature (which is a subset of the 18-protein signature of Ray *et al.*) has the same overall performance. Finally, using more than 20 different classifiers available in the widely-used Weka software package [2], our 5-protein signature has, on average, a smaller prediction error indicating the independence of the classifier and the robustness of this set of biomarkers (i.e. 96% accuracy when predicting AD against non-demented control).

The 6-protein signature is composed of the abundances of IL-1α, IL-3, IL-6, EGF, TNα and G-CSF. We remark that IL-6 was not selected by Ray *et al*. in the preliminary gene selection, and as a consequence it is not part of their 18-protein signature. Recognising that the importance of IL-6 as a biomarker for AD is debatable and that many classifiers do not make use of its abundance to inform decisions, we also present our results of a 5-protein signature that ignores IL-6.

## Results

### Base case–analysis of the performance of randomly selected signatures

Before reporting our experimental results, it was important to understand the worst possible performance results that a set of *k* proteins can have when they are selected at random (from the available 120 proteins under study). We showed results of two experiments that aim at quantifying this. We showed the classification performance of 20 signatures with 18 proteins selected at random with a uniform distribution (obviously, we have selected 18 as is the same number of proteins as the signature proposed by Ray *et al.*). Analogously, we performed the same experiment now constrained to select only six proteins chosen at random (as we will later present comparative results using signatures that only employ 6 and 5 proteins).

The two different collections of 20 sets of randomly generated signatures were chosen using an equal probability for each of the 120 proteins in the set (obviously, not allowing repetitions and constrained to have either 18 or 6 different proteins in total). For this experiment, we decided to use a random forests algorithm (RF) as a base classifier (we are using the algorithm implemented in [3] for reproducibility purposes), generating 150 trees. As the chosen classifier also has a stochastic nature, for each signature we ran 10 experiments with different seeds, and the results we found are quite interesting.

**Figure 1:**
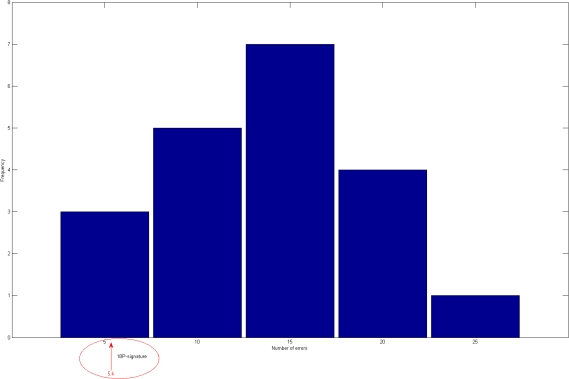
Histograms of the number of errors of the random forest classifier using 20 randomly selected signatures with 18 proteins. The arrow indicates the results under the same conditions of the 18-protein signature proposed by Ray *et al*.

**Figure 2:**
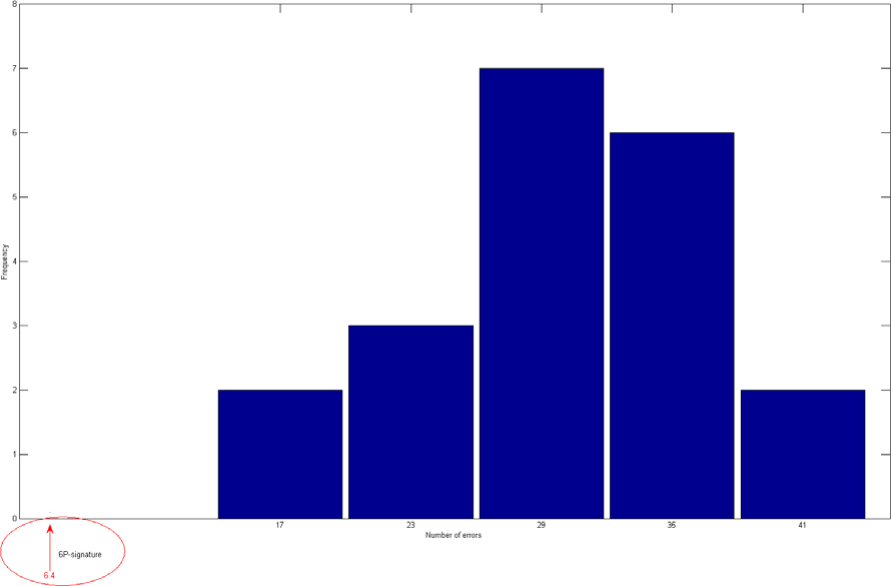
Histograms of the number of errors considering the random forest classifier and the 20 randomly selected signatures with 6 proteins. The arrow indicates the results under the same conditions of our 6-protein signature.

For these twenty 18-protein signatures the average error over the 92 samples considered on the “AD” test set, is 15.13 meaning an 84% effectiveness, see Table 1. For the 6-protein case, an average error of 30.5% was observed meaning that an expected lower value of 67% effectiveness was found, see Table 2. With these results we can infer that the original selection of the 120 genes is quite remarkable for revealing biomarkers for prediction of clinical AD. Since a random selection with a simple, yet robust, classification method allows us to find “good” 18-protein predictor with only a random selection procedure restricted to these 120 proteins. Table 3, Figure 1 and Figure 2 resume the experiment.

**Table 1:** Number of errors from the 18-genes randomly selected signatures on the “AD” validation test set.

Seed Number	S18-1	S18-2	S18-3	S18-4	*S18-5*	S18-6	S18-7	S18-8	*S18-9*	S18-10
76	18	14	11	18	***29***	18	11	10	***4***	10
144	18	15	12	19	***25***	17	13	11	***7***	13
121	18	15	10	22	***25***	19	11	8	***7***	13
83	17	14	11	21	***27***	18	13	12	***6***	15
33	20	18	12	20	***27***	16	11	11	***6***	15
51	15	16	11	21	***26***	17	12	8	***6***	15
162	15	13	13	20	***24***	21	14	8	***7***	13
37	13	14	11	21	***29***	20	10	9	***7***	11
136	17	16	13	22	***23***	20	10	10	***5***	14
60	18	10	11	17	***22***	18	10	9	***7***	15
**Average Error**	**16.9**	**14.5**	**11.5**	**20.1**	***25.7***	**18.4**	**11.5**	**9.6**	***6.2***	**13.4**
**Average Accuracy**	**81.6%**	**84.2%**	**87.5%**	**78.2%**	***72.1%***	**80.0%**	**87.5%**	**89.6%**	***93.3%***	**85.4%**

Seed Number	S18-11	S18-12	S18-13	S18-14	S18-15	S18-16	S18-17	S18-18	S18-19	S18-20
76	17	18	18	16	9	20	7	15	20	11
144	18	22	21	17	9	18	9	16	19	11
121	17	18	20	17	8	15	7	15	22	8
83	16	21	18	16	8	18	10	15	19	13
33	20	22	21	14	8	17	8	15	22	9
51	20	22	22	14	8	20	9	17	22	10
162	19	18	21	16	8	18	7	15	23	10
37	18	21	25	15	7	14	8	15	23	13
136	18	21	17	15	9	18	5	14	23	10
60	19	17	20	13	10	16	9	16	21	12
**Average Error**	**18.2**	**20**	**20.3**	**15.3**	**8.4**	**17.4**	**7.9**	**15.3**	**21.4**	**10.7**
**Average Accuracy**	**80.2%**	**78.3%**	**77.9%**	**83.4%**	**90.9%**	**81.1%**	**91.4%**	**83.4%**	**76.7%**	**88.4%**

The Random forest algorithm was used as classifier. For each signature 10 runs with different seeds were done. We used the WEKA software implementation, and the algorithm was allowed to generate 150 trees. The best and worst signatures are highlighted in bold text. In two cases we found signatures that classify above 90%, comparable with the results of Ray et al. that report on 91% AD predictability as a result of their proposed methodology.

**Table 2:** Number of errors from the 6-genes randomly selected signatures on the “AD” validation test set.

Seed Number	S6-1	S6-2	S6-3	S6-4	S6-5	S6-6	S6-7	S6-8	S6-9	S6-10
76	40	34	20	31	31	32	29	32	24	34
144	40	32	19	34	32	33	30	31	23	33
121	38	37	18	33	35	30	28	32	27	31
83	40	33	19	31	33	34	27	27	24	31
33	41	33	17	35	33	30	27	28	27	29
51	39	33	19	28	34	30	28	28	24	30
162	41	35	19	31	36	34	28	27	26	33
37	40	33	17	32	31	29	27	35	24	32
136	42	36	19	34	34	32	30	34	24	26
60	40	35	17	28	27	31	29	32	23	29
**Average Error**	**40.1**	**34.1**	**18.4**	**31.7**	**32.6**	**31.5**	**28.3**	**30.6**	**24.6**	**30.8**
**Average Accuracy**	**56.4%**	**62.9%**	**80.0%**	**65.5%**	**64.6%**	**65.8%**	**69.2%**	**66.7%**	**73.3%**	**66.5%**

Seed Number	S6-11	S6-12	S6-13	*S6-14*	*S6-15*	S6-16	S6-17	S6-18	S6-19	S6-20
76	32	26	30	***16***	***37***	39	33	34	32	24
144	30	29	36	***17***	***41***	43	32	36	35	24
121	29	25	30	***17***	***41***	37	37	34	35	23
83	31	27	31	***17***	***44***	35	30	35	33	23
33	32	24	32	***17***	***40***	35	35	36	32	23
51	30	25	34	***18***	***41***	38	32	35	33	23
162	33	23	30	***17***	***37***	33	35	36	35	23
37	31	25	31	***17***	***40***	35	32	37	35	22
136	32	29	31	***19***	***43***	35	32	39	34	27
60	31	26	33	***15***	***41***	36	31	38	32	24
**Average Error**	**31.1**	**25.9**	**31.8**	***17***	***40.5***	**36.6**	**32.9**	**36**	**33.6**	**23.6**
**Average Accuracy**	**66.2%**	**71.8%**	**65.4%**	***81.5%***	***56.0%***	**60.2%**	**64.2%**	**60.9%**	**63.5%**	**74.3%**

The Random forest algorithm was used as classifier, for each signature 10 runs with different seeds were done. We used the WEKA software implementation, and the algorithm was allowed to generate 150 trees. The best and worst signatures are highlighted in bold text. This result shows what it is expected, that a 6-signature, when the biomarkers are randomly chosen, is performing significantly worse than the panel of 18 biomarkers selected by Ray *et. al.* Now the best result (81.5%) is worse than the average result of a random 18-signature (86%).

**Table 3:** Random experiments report.

	18-gene random signatures	6-gene random signatures
Average Error	15.14	30.59
Best Signature (average)	6.2	17
Worst Signature (average)	25.7	40.5
Standard Deviation	5.36	6.21
Accuracy Average	**83.5%**	**66.7%**

The table shows the average results of the 20 random signatures for each size, also including the best and worst results and the standard deviation. The accuracy average is calculated considering the error average over the 92 samples of “AD” validation test set.

It is remarkable that by choosing 18 proteins at random we were able to obtain a very good signature, at least for this classifier, under the conditions explained above. Perhaps the reason of obtaining such good signatures is that a smaller number of proteins, that all signatures have in common, is all that it is needed for predictive molecular signature. Figures 1 and 2 show the relation between the considered signatures with 18 and 6 proteins and the random ones.

### Computational studies: Results obtained with four different signatures

We report all the results obtained using a set of 24 classifiers which have been selected from the Weka software suite [3], aiming at sampling different algorithmic methodologies in current practice. These classifiers are applied having as input the four different signatures with the same training set. To ensure reproducibility of our reported methods, no parameter was modified from the classifier's default setting from Weka's downloaded code. In this way we were not biasing the experiment with *ad hoc* parameter selection and we ensure the complete reproducibility of our claims. We are also aware that better results are possible when adjusting the parameters of each classifier considering only the samples of the training set. Nevertheless, with these tests our objective is to show the robustness of our methods to discovery biomarkers, by showing the independence of the signature performance from the selected classifier.

It is interesting to note that the mathematical model and algorithms we have used have pointed at Interleukin-6 and included it in the 10-protein signature. It is well known that IL-6 with other cytokines have been the subject of many studies of biomarkers for Alzheimer's disease [4]–[6]. Using an integrative bioinformatic approach, described in the next sections, we draw our attention to a smaller signature. The 6-protein signature was obtained by the analysis of the protein-relation graph and interestingly enough, IL-6 is also included in this new core signature. Finally, in the 5-protein signature, IL-6 is excluded to provide another comparison and the five proteins now become a proper subset of the 18 original proteins uncovered by Ray *et al.* Table 4 presents the genes included in each signature, indicating the protein name, Entrez GeneID and official name.

**Table 4:** Protein name for each signature used in the computational experiment.

Protein Name	Entrez GeneID	Official gene name provided by HUGO Gene Nomenclature Committee (HGNC)	In signature			
			18	10	6	5
ANG-2	285	angiopoietin 2	x			
CCL5/RANTES	6352	chemokine (C-C motif) ligand 5	x			
CCL7/MCP-3	6354	chemokine (C-C motif) ligand 7	x	x		
CCL15/MIP-1δ	6359	chemokine (C-C motif) ligand 15	x	x		
CCL18/PARC	6362	chemokine (C-C motif) ligand 18 (pulmonary and activation-regulated)	x			
CXCL8/IL-8	3576	interleukin 8	x			
EGF	1950	epidermal growth factor (beta-urogastrone)	x	x	x	x
G-CSF	1440	colony stimulating factor 3 (granulocyte)	x	x	x	x
GDNF	2668	glial cell derived neurotrophic factor	x			
ICAM-1	3383	intercellular adhesion molecule 1 (CD54), human rhinovirus receptor	x			
IGFBP-6	3489	insulin-like growth factor binding protein 6	x			
IL-1α	3552	interleukin 1, alpha	x	x	x	x
IL-3	3562	interleukin 3 (colony-stimulating factor, multiple)	x	x	x	x
IL-6	3569	interleukin 6 (interferon, beta 2)		x	x	
IL-11	3589	interleukin 11	x	x		
M-CSF	1435	colony stimulating factor 1 (macrophage)	x			
PDGF-BB	5155	platelet-derived growth factor beta polypeptide (simian sarcoma viral (v-sis) oncogene homolog)	x	x		
TNF-α	7124	tumor necrosis factor (TNF superfamily, member 2)	x	x	x	x
TRAIL R4	8793	tumor necrosis factor receptor superfamily, member 10d, decoy with truncated death domain	x			

Tables 5, 6, 7 and 8 show the results of the 24 classifiers for all the signatures considered. The classifiers marked with a star have a random component; therefore the average of ten runs with different seeds is reported. Finally, Tables 9 and 10 summarize the results.

**Table 5:** Report of the results of the 24 classifiers when using the 18-Protein biomarker.

Classifier	Grand Total	OVERALL (“AD”+“MCI”)		Test Set “AD”		Test Set “MCI”	
		AD Er.	NAD Er.	AD Er.	NAD Er.	AD Er.	NAD Er.
Dataset size	139	64	75	42	50	22	25
**PAM**	21	7	14	4	6	3	8
**SMO**	20	5	15	2	6	3	9
**Simple Logistic**	25	10	15	5	6	5	9
**Logistic**	27	11	16	6	7	5	9
Multilayer Perceptron*	21.7	10.1	11.6	4	3.3	6.1	8.3
**Bayes Net**	27	7	20	3	7	4	13
**Naïve Bayes**	23	4	19	1	5	3	14
**Naïve Bayes Simple**	23	4	19	1	5	3	14
**Naïve Bayes Up**	23	4	19	1	5	3	14
**IB1**	21	5	16	2	3	3	13
**Ibk**	21	5	16	2	3	3	13
**Kstar**	28	5	23	2	11	3	12
**LWL**	28	15	13	5	3	10	10
**AdaBoost**	23	9	14	4	3	5	11
**ClassViaRegression**	28	14	14	5	4	9	10
Decorate*	23.1	7.9	15.2	3.3	5.2	4.6	10
**MultiClass Classifier**	27	11	16	6	7	5	9
Random Committee*	26.1	10.1	16	4.4	5.5	5.7	10.5
**j48**	24	13	11	3	2	10	9
**LMT**	25	10	15	5	6	5	9
**NBTree**	26	13	13	5	4	8	9
**Part**	25	14	11	7	2	7	9
Random Forest*	24.3	9.3	15	4.1	4	5.2	11
**Ordinal Classifier**	24	13	11	3	2	10	9
**Average**	**24.34**	**9.02**	**15.33**	**3.66**	**4.79**	**5.36**	**10.53**
**Agreement (%)**	**82%**	**86%**	**80%**	**91%**	**90%**	**76%**	**58%**

18-Protein Signature (Ray *et al.*)

**Table 6:** Report of the results of the 24 classifiers when using the 10-Protein biomarker.

10-Protein Signature							
Classifier	Grand Total	OVERALL (“AD”+“MCI”)		Test Set “AD”		Test Set “MCI”	
		AD Er.	NAD Er.	AD Er.	NAD Er.	AD Er.	NAD Er.
Dataset size	139	64	75	42	50	22	25
**PAM**	23	5	18	3	8	2	10
**SMO**	23	7	16	2	6	5	10
**Simple Logistic**	23	4	19	1	8	3	11
**Logistic**	24	6	18	1	9	5	9
Multilayer Perceptron*	21.8	4.9	16.9	1.2	6.9	3.7	10
**Bayes Net**	28	7	21	1	8	6	13
**Naïve Bayes**	31	6	25	2	12	4	13
**Naïve Bayes Simple**	31	6	25	2	12	4	13
**Naïve Bayes Up**	31	6	25	2	12	4	13
**IB1**	28	6	22	3	9	3	13
**Ibk**	28	6	22	3	9	3	13
**Kstar**	39	3	36	0	18	3	18
**LWL**	28	15	13	5	3	10	10
**AdaBoost**	22	4	18	1	8	3	10
**ClassViaRegression**	23	8	15	1	5	7	10
Decorate*	25.1	6.7	18.4	1.6	8	5.1	10.4
**MultiClass Classifier**	24	6	18	1	9	5	9
Random Committee*	25.8	9.9	15.9	3.3	6.4	6.6	9.5
**j48**	22	11	11	3	2	8	9
**LMT**	37	17	20	8	12	9	8
**NBTree**	19	13	6	5	3	8	3
**Part**	21	10	11	3	2	7	9
Random Forest*	23.9	9.4	14.5	2.7	5	6.7	9.5
**Ordinal Classifier**	22	11	11	3	2	8	9
**Average**	**25.99**	**7.83**	**18.15**	**2.45**	**7.64**	**5.38**	**10.52**
**Agreement (%)**	**81%**	**88%**	**76%**	**94%**	**85%**	**76%**	**58%**

**Table 7:** Report of the results of the 24 classifiers when using the 6-Protein biomarker.

6-Protein Signature							
Classifier	Grand Total	OVERALL (“AD”+“MCI”)		Test Set “AD”		Test Set “MCI”	
		AD Er.	NAD Er.	AD Er.	NAD Er.	AD Er.	NAD Er.
Dataset size	139	64	75	42	50	22	25
**PAM**	20	8	12	1	3	7	9
**SMO**	20	9	11	2	2	7	9
***Simple Logistic***	*18*	*4*	*14*	*0*	*4*	*4*	*10*
**Logistic**	21	4	17	0	7	4	10
Multilayer Perceptron*	25.6	3.2	22.4	0.4	9	2.8	13.4
**Bayes Net**	22	8	14	3	4	5	10
**Naïve Bayes**	23	8	15	2	5	6	10
**Naïve Bayes Simple**	24	9	15	3	5	6	10
**Naïve Bayes Up**	23	8	15	2	5	6	10
**IB1**	33	9	24	3	11	6	13
**Ibk**	33	9	24	3	11	6	13
**Kstar**	33	6	27	1	13	5	14
**LWL**	29	16	13	6	3	10	10
**AdaBoost**	27	11	16	3	6	8	10
**ClassViaRegression**	23	10	13	3	6	7	7
Decorate*	24.7	9.8	14.9	2.4	4.8	7.4	10.1
**MultiClass Classifier**	21	4	17	0	7	4	10
Random Committee*	26.6	11.5	15.1	3.1	5.6	8.4	9.5
**j48**	24	10	14	2	5	8	9
***LMT***	*18*	*4*	*14*	*0*	*4*	*4*	*10*
**NBTree**	21	10	11	1	2	9	9
**Part**	27	13	14	3	5	10	9
Random Forest*	25.6	11.8	13.8	2.6	4.4	9.2	9.4
**Ordinal Classifier**	24	10	14	2	5	8	9
**Average**	**24.44**	**8.60**	**15.84**	**2.02**	**5.70**	**6.58**	**10.14**
**Agreement (%)**	**82%**	**87%**	**79%**	**95%**	**89%**	**70%**	**59%**

Using this biomarker it is notable the effectiveness of predicting AD on the “AD” test set using simple classifiers as simple logistic or LMT (Logistic Model Tree) or even the same classifier used in [1] (PAM).

**Table 8:** Report of the results of the 24 classifiers when using the 5-Protein biomarker.

5-Protein Signature							
Classifier	Grand Total	OVERALL (“AD”+“MCI”)		Test Set “AD”		Test Set “MCI”	
		AD Er.	NAD Er.	AD Er.	NAD Er.	AD Er.	NAD Er.
Dataset size	139	64	75	42	50	22	25
**PAM**	21	10	11	3	2	7	9
**SMO**	19	8	11	2	2	6	9
***Simple Logistic***	***18***	***4***	***14***	***0***	***4***	***4***	***10***
**Logistic**	20	4	16	0	6	4	10
Multilayer Perceptron*	21.6	5.3	16.3	0.7	5.2	4.6	11.1
**Bayes Net**	21	4	17	1	5	3	12
**Naïve Bayes**	19	5	14	1	2	4	12
**Naïve Bayes Simple**	20	5	15	1	3	4	12
**Naïve Bayes Up**	19	5	14	1	2	4	12
**IB1**	30	10	20	3	7	7	13
**Ibk**	30	10	20	3	7	7	13
**Kstar**	26	8	18	3	7	5	11
**LWL**	29	16	13	6	3	10	10
**AdaBoost**	31	3	28	1	11	2	17
**ClassViaRegression**	24	5	19	1	7	4	12
Decorate*	21.8	8.7	13.1	1.7	3.9	7	9.2
**MultiClass Classifier**	20	4	16	0	6	4	10
Random Committee*	26.1	10.9	15.2	3.1	5.1	7.8	10.1
**j48**	24	10	14	2	5	8	9
***LMT***	***18***	***4***	***14***	***0***	***4***	***4***	***10***
**NBTree**	21	10	11	1	2	9	9
**Part**	27	13	14	3	5	10	9
Random Forest*	26.2	12.1	14.1	3.2	4.9	8.9	9.2
**Ordinal Classifier**	24	10	14	2	5	8	9
**Average**	**23.20**	**7.71**	**15.49**	**1.78**	**4.75**	**5.93**	**10.73**
**Agreement (%)**	**83%**	**88%**	**79.4%**	**96%**	**90%**	**73%**	**57%**

Removing IL-6 from the biomarker set we have a small gain in predicting AD in both data set, if compared to the 6-protein signature. In this case, the prediction of AD on the “AD” test set achieves an average of 96% without dropping the accuracy of the prediction of NonAD.

The results of our 5-protein signature are reported in Table 8. When considering the “AD” test set, *average* results (over 24 classifiers) are obtained by the 5-protein signature, 96% when predicting AD and 90% when predicting non-demented control. It is also worth mentioning that there are four different classifiers achieving almost 100% accuracy (i.e. having a number of errors smaller or equal to 1) for predicting AD on the “AD” test set. These results are achieved without losing accuracy when predicting non-demented controls on the same dataset.

In Table 9, a feature of the experiments it is worth commenting: all the signatures drop at least 30% in accuracy when considering the “MCI” dataset. This is understandable since the classifiers have no sample labelled “MCI” in the training set.

**Table 9:** Average results for each signature over 24 classifiers.

	Size	Overall	Overall (“AD”+“MCI”)		Test set “AD”		Test set “MCI”	
			AD Er.	NAD Er.	AD Er.	NAD Er.	AD Er.	NAD Er.
		139	64	75	42	50	22	25
**18 protein Sig.**	Error Avg	24.34	9.02	15.33	3.66	4.79	5.36	10.53
	Agr %	82%	86%	**80%**	91%	**90%**	**76%**	58%
			82%		91%		**66%**	
**10 protein Sig.**	Error Avg	25.98	7.83	18.15	2.45	7.64	5.38	10.52
	Agr %	81%	**88%**	76%	94%	85%	**76%**	58%
			81%		89%		**66%**	
**6 protein Sig.**	Error Avg	24.44	8.60	15.84	2.02	5.70	6.58	10.14
	Agr %	82%	87%	79%	95%	89%	70%	**59%**
			82%		92%		64%	
**5 protein Sig.**	Error Avg	23.20	7.71	15.49	1.78	4.75	5.93	10.73
	Agr %	**83%**	**88%**	79%	**96%**	**90%**	73%	57%
			**83%**		**93%**		65%	

For each signature the average number of errors is reported and the percentage agreement is calculated over each specific population. The best results are highlighted in bold text.

The best overall result, considering both test sets, is obtained by the 6-protein and 5-protein signatures. They present 18 errors and in both signatures this result is obtained twice when using the LMT and Simple Logistic classifiers (Tables 7 and 8).

In Table 10, the standard deviations of the number of errors are almost constant for all signatures, in all datasets. This reinforces our previous claim, the poor performance of the signatures on the “MCI” dataset is related to the fact that the signatures were not trained to identify between AD and MCI.

**Table 10:** The standard deviation of each test is shown on this table.

	Overall (“AD”+“MCI”)		Test set AD		Test set MCI	
	AD Er.	NAD Er.	AD Er.	NAD Er.	AD Er.	NAD Er.
**18 protein Sig.**	3.580	3.022	1.692	2.087	2.430	1.982
**10 protein Sig.**	3.546	6.127	1.721	3.893	2.214	2.729
**6 protein Sig.**	3.165	4.218	1.419	2.798	2.024	1.625
**5 protein Sig.**	3.520	3.668	1.433	2.175	2.326	1.906

All the signatures show a very similar behaviour with a small standard deviation.

To present the experiment results in another form, we compared the performance of each signature in each test. Table 11 presents the comparison between the signatures when considering *all* the test sets (“AD”+“MCI”) totalling 139 samples. It is remarkable that the 5-protein signature not only has a better average performance, but also presents the best result on 16 of the 24 algorithms used for classification (the number of errors highlighted in bold text indicates the best performance for this particular classifier).

**Table 11:** Number of errors for each classifier when considering both test sets together (139 samples).

Method	Overall errors			
	*18*	*10*	*6*	*5*
Simple Logistic	25	25	18	**18**
LMT	25	25	**18**	**18**
Logistic	27	24	21	**20**
MultiClass Classifier	27	24	21	**20**
Bayes Net	27	28	22	**21**
NBTree	26	23	**21**	**21**
Naïve Bayes	23	30	23	**19**
Naïve Bayes Up.	23	30	23	**19**
ClassViaRegression	28	25	**23**	24
Naïve Bayes Simple	23	30	24	**20**
Kstar	28	41	33	**26**
Decorate	23.1	28.3	24.7	**21.8**
SMO	20	23	20	**19**
Multilayer Perceptron	21.7	21.8	25.6	**21.6**
PAM	21	22	**20**	21
Random Committee	**26.1**	26.3	26.6	**26.1**
j48	**24**	**24**	**24**	**24**
Ordinal Class Classifier	**24**	**24**	**24**	**24**
LWL	**28**	**28**	29	29
Random Forest	**24.3**	**24.3**	25.6	26.2
Part	**25**	30	27	27
AdaBoost	**23**	31	27	31
IB1	**21**	28	33	30
Ibk	**21**	28	33	30
Average	24.342	26.821	24.438	**23.196**
Agreement %	82%	81%	82%	**83%**

The signature with the best performance on each classifier is highlighted in bold text.

In Table 12, the same comparison is made but only considering the “AD” test set. Once again, it is possible to visualize the performance of the 5-protein signature, obtaining not only the best average result but also the best individual results, presenting 3 errors on 3 occasions.

**Table 12:** Number of errors for each classifier when considering the “AD” test set (92 samples).

Method	“AD” test set			
	*18*	*10*	*6*	*5*
NBTree	9	8	**3**	**3**
Simple Logistic	11	9	**4**	**4**
LMT	11	20	**4**	**4**
Logistic	13	10	7	**6**
MultiClass Classifier	13	10	7	**6**
PAM	10	11	**4**	5
SMO	8	8	**4**	**4**
Naïve Bayes	6	14	7	**3**
Naïve Bayes Up.	6	14	7	**3**
Bayes Net	10	9	7	**6**
Decorate	8.5	9.6	7.2	**5.6**
Naïve Bayes Simple	6	14	8	**4**
Kstar	13	18	14	**10**
Multilayer Perceptron	7.3	8.1	9.4	**5.9**
Random Committee	9.9	9.7	8.7	**8.2**
ClassViaRegression	9	**6**	9	8
Part	9	**5**	8	8
Random Forest	8.1	7.7	**7**	8.1
LWL	**8**	**8**	9	9
j48	**5**	**5**	7	7
Ordinal Class Classifier	**5**	**5**	7	7
AdaBoost	**7**	9	9	12
IB1	**5**	12	14	10
Ibk	**5**	12	14	10
Average	8.45	10.09	7.72	**6.53**
Agreement %	91%	89%	92%	**93%**

The signature with the best performance on each classifier is highlighted in bold text.

Finally, Table 13 presents the same analysis for the “MCI” test set. In this case the most remarkable observation is the lack of quality to predict MCI-AD. The improved performance of the largest signatures is related to the fact that the signatures have more genes, and because they were not trained to distinguish between MCI patients, the use of more proteins allows a slightly better performance. Nevertheless, even the best signature for this case (a 10-protein signature) presents a poor performance when compared with the previous results.

**Table 13:** Number of errors for each classifier when considering the “MCI” test set (47 samples).

Method	“MCI” test set			
	*18*	*10*	*6*	*5*
ClassViaRegression	19	17	**14**	16
Bayes Net	17	19	**15**	**15**
j48	19	**17**	**17**	**17**
Ordinal Class Classifier	19	**17**	**17**	**17**
Naïve Bayes	17	17	**16**	**16**
Naïve Bayes Simple	17	17	**16**	**16**
Naïve Bayes Up.	17	17	**16**	**16**
Simple Logistic	**14**	**14**	**14**	**14**
Logistic	**14**	**14**	**14**	**14**
LWL	**20**	**20**	**20**	**20**
MultiClass Classifier	**14**	**14**	**14**	**14**
LMT	**14**	17	**14**	**14**
NBTree	17	**11**	18	18
Kstar	**15**	21	19	16
Multilayer Perceptron	14.4	**13.7**	16.2	15.7
Random Committee	16.2	**16.1**	17.9	17.9
Decorate	**14.6**	15.5	17.5	16.2
Random Forest	**16.2**	**16.2**	18.6	18.1
AdaBoost	16	**13**	18	19
Part	**16**	**16**	19	19
IB1	**16**	**16**	19	20
Ibk	**16**	**16**	19	20
SMO	**12**	15	16	15
PAM	**11**	12	16	16
Average	16.29	16.11	**16.78**	**16.77**
Agreement %	65%	66%	**64%**	**64%**

The signature with the best performance on each classifier is highlighted in bold text.

## Discussion

In conclusion, it is clear that the experiment performed by Ray *et al.* provided an extremely useful dataset for the identification of Alzheimer's disease biomarkers. We have uncovered a robust 5-protein signature with near 97% of accuracy to predict AD against non-demented controls using their data. Our signature has less than one third of the proteins than the one proposed in the original paper, and at least the same level of prediction performance.

The next step on this important quest is to set up an independent experimental procedure that now considers samples with mild cognitive impairment (but without AD) in the training set. We do not agree with the methodology of using a training set without MCI to select biomarkers to differentiate between AD and MCI [1]. This has not been done and warrants further investigation. Only in this way we can uncover useful biomarkers to discriminate between AD and MCI.

On the positive side, our methods reveal the true predictive potential of testing for Alzheimer's disease using this panel of signalling proteins. We also believe that our methods show promise and warrant their application in other settings. It is clear that Alzheimer researchers can benefit directly from our identification of more robust biomarkers. The method is revealed to be useful, simple yet very powerful, and warrants its application in other multifactorial diseases.

## Methods

Our methodology consisted of the application of an integrative data analysis method. We used four steps: a) abundance quantization, b) feature selection, c) literature analysis, d) selection of a classifier algorithm which is independent of the feature selection process. These steps were performed without using any of the test datasets. For the first two steps, we used the application of Fayyad and Irani's discretization algorithm [7] for selection and quantization, which in turn creates an instance of the *(alpha-beta)-k-Feature Set problem* [8]–[10]. Fayyad and Irani's method filtered only 14 out of 120 proteins of the training set (i.e. those proteins for which no threshold was selected were filtered out). After quantization, samples 7, 43 (AD, “Alzheimer's Disease”) and 48 (NDC, “Nondemented Control”) of the training set were “in conflict”, which means that they have quantized values (for all 14 proteins selected) which are the same although they belong to different classes. These conflicts are then removed, *i.e.* the three samples of the training set are eliminated and we apply our algorithms to the remaining 80 samples of the training set. Numerical solution of the *(alpha-beta)-k-Feature Set problem* led to the selection of only 10 proteins, Table 4. For a detailed explanation of the methods and other applications, readers can check our referenced publications and references therein [11]–[13].

To guarantee the reproduction of all our experiments, we use algorithms from the Weka Package [3] as classifiers. All the classifiers were used with the default parameters; we are convinced that better results could be found if adjustments are made in each classifier (considering only its result over the training set).

**Figure 3:**
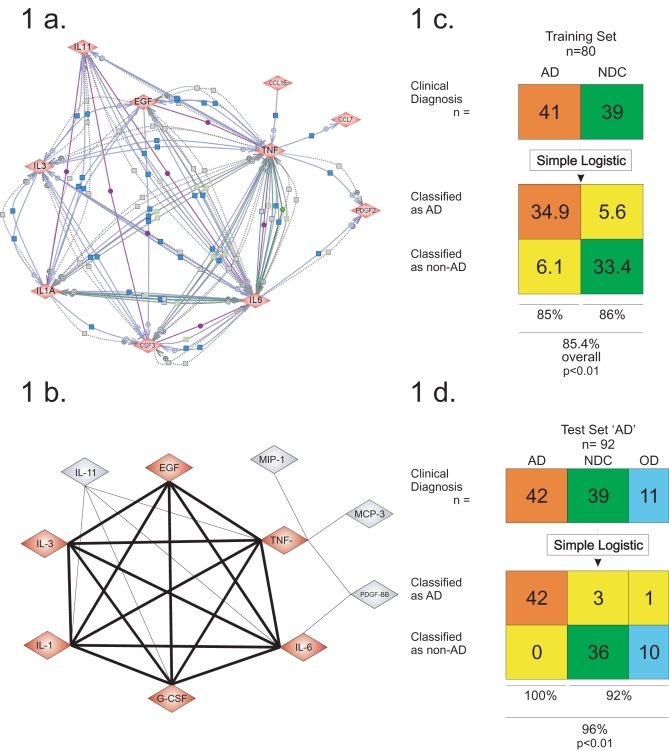
Classification and prediction of clinical Alzheimer's diagnosis in subjects with Alzheimer's disease. (a) An undirected graph, where each node corresponds a different protein belonging to the 10-protein signature we identified; each edge indicates the existence of a direct relation as obtained by searching the PubMed database, (using the Pathway Studio software). (b) Identification of the maximum clique of the graph, uncovering a robust 6-protein signature; each node on the clique has a direct relation with each other. Simple Logistic was used to classify and predict Alzheimer's (AD) and non-Alzheimer's class, in the training set (c), the blinded test set ‘AD’ (d). All the results are shown in a confusion matrix, for the training set a 10-fold cross-validation was applied 10 times, in both cases Simple Logistic was used with the default parameters of Weka package. All the p-values were calculated using the Fisher exact test.

The first signature we uncovered contains 10 proteins, see Table 4. Using the Pathway Studio software [3], we generated an undirected graph of the known ‘direct relations’ of these 10 proteins. Each node in the graph corresponds to a protein and an edge exists if the Pathway Studio software produced a ‘direct relation’, indicating important association already observed in the life sciences literature. On this graph we looked for its maximum clique (Fig. 3a). We denote this graph as *G = (V,E)*. Each vertex in *V* has a one-to-one correspondence with a protein. Each pair of vertices are connected by an edge in *E,* if and only if, there are many direct relations between the proteins reported in the literature. A clique in *G* is a subset *X* of *V* such that its induced graph *G[X]* is complete. In other words, we are looking for the maximum subset of proteins, in which all pairs of proteins already have a direct relationship identified between them, thus we consider this set the *core* of our 10-protein signature (this core has the 6-proteins listed above, see Fig. 3b).

Our first benchmark test for this 6-protein signature was done using Simple Logistic (SL) [14], perhaps the simplest classifier from the Weka software suite. With our 6-protein signature, SL had a performance of 86% after applying 10 times 10-fold cross-validation over the training set (Fig. 3c). When considering the “AD” test set, our 6-protein signature with SL was able to make a classification with 97% of accuracy. For AD samples we achieved 100% positive agreement and for NDC samples a 92% negative agreement (Fig. 3d).

When using the second test set (labelled “MCI”), that includes samples that had an initial diagnosis of mild cognitive impairment, the performance of all signatures increases the number of errors. It is reasonable to expect that our very trimmed classifiers are going to have some degradation of performance, as they have not been trained to distinguish confirmed AD samples from those that have MCI. When using the same signature to differentiate between AD and other samples of MCI patients, the occurrence of more errors is an expected outcome (Table 9). In spite of this fact, the overall performance of all signatures seems very robust.
